# Crystal structure of [1,1′-biphen­yl]-2,2′-dicarbo­nitrile

**DOI:** 10.1107/S2056989015009561

**Published:** 2015-05-30

**Authors:** Gihaeng Kang, Tae Ho Kim, Youngeun Jeon, Jineun Kim

**Affiliations:** a Department of Chemistry and Research Institute of Natural Sciences, Gyeongsang National University, Jinju 660-701, Republic of Korea

**Keywords:** crystal structure, biphen­yl, π–π contacts

## Abstract

The complete mol­ecule of the title compound, C_14_H_8_N_2_, is generated by a twofold rotation axis located at the midpoint of the biphenyl C—C bond. The dihedral angle between the symmetry-related phenyl rings is 46.16 (3)°. In the crystal, mol­ecules are linked by slipped parallel π–π inter­actions [centroid–centroid distance = 3.9451 (7) Å, normal distance = 3.6293 (5) Å, slippage 1.547 Å], forming columns along the *b*-axis direction.

## Related literature   

The title compound has been used as a reactant for phthalocyanine synthesis (Shimizu *et al.*, 2011 ▸, 2014 ▸). Related crystal structures were reported by Furukawa *et al.* (2008 ▸) and Paek *et al.* (1989 ▸). For synthetic details, see: Wu *et al.* (2007 ▸).
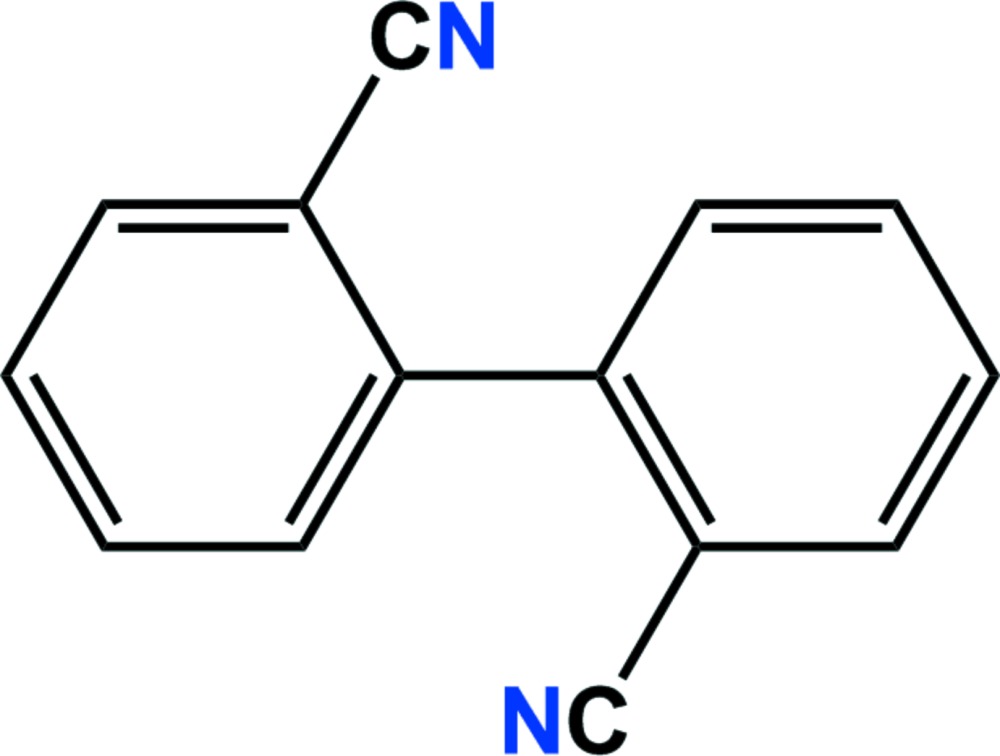


## Experimental   

### Crystal data  

C_14_H_8_N_2_
*M* *_r_* = 204.22
Monoclinic, 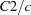

*a* = 15.7839 (9) Å
*b* = 3.9451 (2) Å
*c* = 16.6079 (9) Å
β = 101.630 (3)°
*V* = 1012.93 (9) Å^3^
*Z* = 4
Mo *K*α radiation
μ = 0.08 mm^−1^
*T* = 173 K
0.43 × 0.12 × 0.06 mm

### Data collection  

Bruker APEXII CCD diffractometer
Absorption correction: multi-scan (*SADABS*; Bruker, 2009 ▸) *T*_min_ = 0.966, *T*_max_ = 0.995
4708 measured reflections
1157 independent reflections
988 reflections with *I* > 2σ(*I*)
*R*_int_ = 0.030

### Refinement  

*R*[*F* ^2^ > 2σ(*F* ^2^)] = 0.042
*wR*(*F* ^2^) = 0.116
*S* = 1.09
1157 reflections
73 parameters
H-atom parameters constrained
Δρ_max_ = 0.21 e Å^−3^
Δρ_min_ = −0.24 e Å^−3^

### 

Data collection: *APEX2* (Bruker, 2009 ▸); cell refinement: *SAINT* (Bruker, 2009 ▸); data reduction: *SAINT*; program(s) used to solve structure: *SHELXTL* (Sheldrick, 2008 ▸); program(s) used to refine structure: *SHELXTL*; molecular graphics: *DIAMOND* (Brandenburg, 2010 ▸); software used to prepare material for publication: *SHELXTL*.

## Supplementary Material

Crystal structure: contains datablock(s) global, I. DOI: 10.1107/S2056989015009561/wm5163sup1.cif

Structure factors: contains datablock(s) I. DOI: 10.1107/S2056989015009561/wm5163Isup2.hkl

Supporting information file. DOI: 10.1107/S2056989015009561/wm5163Isup3.cml

**Fig. 1.**
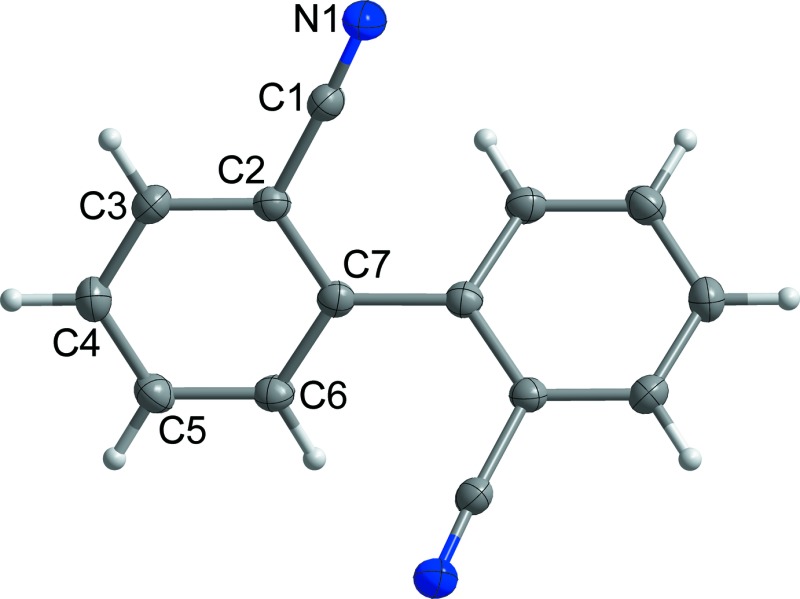
The molecular structure of the title compound with the atom-numbering scheme. Displacement ellipsoids are drawn at the 50% probability level. H atoms are shown as small spheres of arbitrary radius. Symmetry-related atoms (not labelled) are generated by symmetry code x+1, y, -z+1/2.

**Fig. 2.**
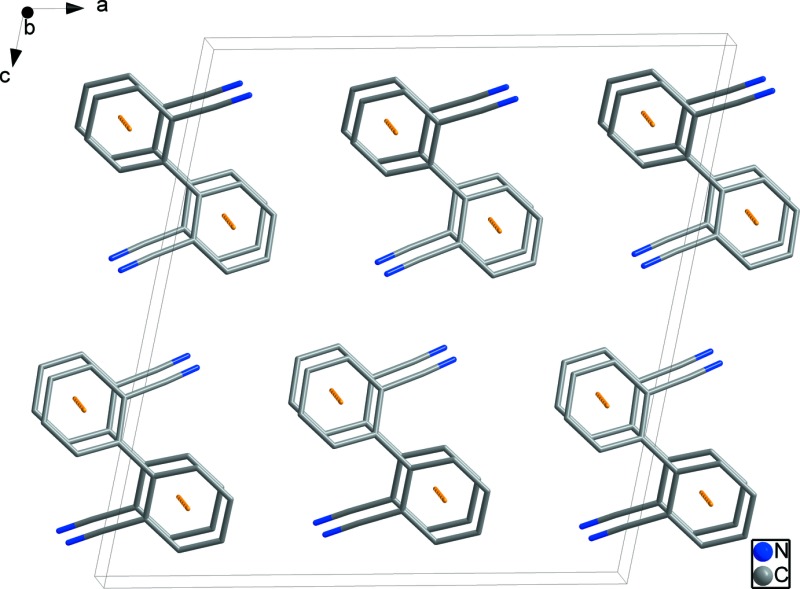
Crystal packing viewed along the b axis. The intermolecular π–π interactions between the phenyl ring systems [Cg1···Cg1i, 3.9451 (7) Å; Cg1 is the centroid of the C2···C7 ring; symmetry code (i): x, y - 1, z] are shown as dashed lines. They link molecules into columns along [010].

Click here for additional data file.x y z . DOI: 10.1107/S2056989015009561/wm5163fig1.tif
The mol­ecular structure of the title compound with the atom-numbering scheme. Displacement ellipsoids are drawn at the 50% probability level. H atoms are shown as small spheres of arbitrary radius. Symmetry-related atoms (not labelled) are generated by symmetry code *x* + 1, *y*, −*z* + 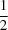.

b Cg Cg i Cg x y z . DOI: 10.1107/S2056989015009561/wm5163fig2.tif
Crystal packing viewed along the *b* axis. The inter­molecular π–π inter­actions between the phenyl ring systems [*Cg*1⋯*Cg*1^i^, 3.9451 (7) Å; *Cg*1 is the centroid of the C2⋯C7 ring; symmetry code (i): *x*, *y* − 1, *z*] are shown as dashed lines. They link mol­ecules into columns along [010].

CCDC reference: 1401615

Additional supporting information:  crystallographic information; 3D view; checkCIF report

## supplementary crystallographic information

### S1. Experimental

The title compound was prepared by Suzuki coupling reaction of 2-bromobenzonitrile and 2-cyanophenyl boronic acid in acetonitrile (Wu *et al.*, 2007). Slow evaporation of a solution in acetone/ethyl acetate gave single crystals suitable for X-ray analysis.

### S2. Refinement

All H-atoms were positioned geometrically and refined using a riding model with *d*(C—H) = 0.95 Å, *U*_iso_(H) = 1.2*U*_eq_(C).

### Crystal data

**Table:**

C_14_H_8_N_2_	*F*(000) = 424
*M**_r_* = 204.22	*D*_x_ = 1.339 Mg m^−^^3^
Monoclinic, *C*2/*c*	Mo *K*α radiation, λ = 0.71073 Å
*a* = 15.7839 (9) Å	Cell parameters from 1375 reflections
*b* = 3.9451 (2) Å	θ = 3.3–27.5°
*c* = 16.6079 (9) Å	µ = 0.08 mm^−^^1^
β = 101.630 (3)°	*T* = 173 K
*V* = 1012.93 (9) Å^3^	Block, colourless
*Z* = 4	0.43 × 0.12 × 0.06 mm

### Data collection

**Table:**

Bruker APEXII CCD diffractometer	1157 independent reflections
Radiation source: fine-focus sealed tube	988 reflections with *I* > 2σ(*I*)
Graphite monochromator	*R*_int_ = 0.030
φ and ω scans	θ_max_ = 27.6°, θ_min_ = 3.3°
Absorption correction: multi-scan (*SADABS*; Bruker, 2009)	*h* = −20→20
*T*_min_ = 0.966, *T*_max_ = 0.995	*k* = −5→1
4708 measured reflections	*l* = −21→19

### Refinement

**Table:**

Refinement on *F*^2^	Primary atom site location: structure-invariant direct methods
Least-squares matrix: full	Secondary atom site location: difference Fourier map
*R*[*F*^2^ > 2σ(*F*^2^)] = 0.042	Hydrogen site location: inferred from neighbouring sites
*wR*(*F*^2^) = 0.116	H-atom parameters constrained
*S* = 1.09	*w* = 1/[σ^2^(*F*_o_^2^) + (0.0584*P*)^2^ + 0.5212*P*] where *P* = (*F*_o_^2^ + 2*F*_c_^2^)/3
1157 reflections	(Δ/σ)_max_ < 0.001
73 parameters	Δρ_max_ = 0.21 e Å^−^^3^
0 restraints	Δρ_min_ = −0.24 e Å^−^^3^

### Special details

**Table:**

Geometry. All e.s.d.'s (except the e.s.d. in the dihedral angle between two l.s. planes) are estimated using the full covariance matrix. The cell e.s.d.'s are taken into account individually in the estimation of e.s.d.'s in distances, angles and torsion angles; correlations between e.s.d.'s in cell parameters are only used when they are defined by crystal symmetry. An approximate (isotropic) treatment of cell e.s.d.'s is used for estimating e.s.d.'s involving l.s. planes.
Refinement. Refinement of *F*^2^ against ALL reflections. The weighted *R*-factor *wR* and goodness of fit *S* are based on *F*^2^, conventional *R*-factors *R* are based on *F*, with *F* set to zero for negative *F*^2^. The threshold expression of *F*^2^ > σ(*F*^2^) is used only for calculating *R*-factors(gt) *etc*. and is not relevant to the choice of reflections for refinement. *R*-factors based on *F*^2^ are statistically about twice as large as those based on *F*, and *R*- factors based on ALL data will be even larger.

### Fractional atomic coordinates and isotropic or equivalent isotropic displacement parameters (Å^2^)

**Table:**

	*x*	*y*	*z*	*U*_iso_*/*U*_eq_	
N1	0.58879 (7)	0.3650 (3)	0.08819 (7)	0.0323 (3)	
C1	0.53155 (8)	0.2522 (3)	0.11172 (7)	0.0233 (3)	
C2	0.45640 (7)	0.1152 (3)	0.13725 (7)	0.0206 (3)	
C3	0.37963 (8)	0.1099 (3)	0.07828 (7)	0.0244 (3)	
H3	0.3791	0.1894	0.0242	0.029*	
C4	0.30457 (8)	−0.0108 (3)	0.09854 (8)	0.0273 (3)	
H4	0.2522	−0.0139	0.0586	0.033*	
C5	0.30609 (8)	−0.1274 (3)	0.17741 (8)	0.0257 (3)	
H5	0.2545	−0.2117	0.1914	0.031*	
C6	0.38225 (8)	−0.1224 (3)	0.23640 (7)	0.0231 (3)	
H6	0.3819	−0.2037	0.2902	0.028*	
C7	0.45930 (7)	−0.0004 (3)	0.21832 (7)	0.0196 (3)	

### Atomic displacement parameters (Å^2^)

**Table:**

	*U*^11^	*U*^22^	*U*^33^	*U*^12^	*U*^13^	*U*^23^
N1	0.0275 (6)	0.0450 (7)	0.0253 (6)	−0.0066 (5)	0.0075 (4)	0.0013 (5)
C1	0.0244 (6)	0.0277 (7)	0.0176 (6)	−0.0017 (5)	0.0034 (4)	−0.0014 (5)
C2	0.0208 (6)	0.0225 (6)	0.0194 (6)	−0.0003 (4)	0.0059 (4)	−0.0015 (4)
C3	0.0258 (6)	0.0290 (7)	0.0180 (6)	−0.0002 (5)	0.0037 (5)	0.0003 (4)
C4	0.0214 (6)	0.0331 (7)	0.0255 (7)	−0.0011 (5)	0.0000 (5)	−0.0016 (5)
C5	0.0206 (6)	0.0286 (6)	0.0286 (7)	−0.0028 (5)	0.0069 (5)	−0.0006 (5)
C6	0.0245 (6)	0.0239 (6)	0.0216 (6)	−0.0017 (5)	0.0068 (5)	0.0020 (4)
C7	0.0205 (6)	0.0187 (6)	0.0199 (6)	0.0012 (4)	0.0044 (5)	−0.0013 (4)

### Geometric parameters (Å, º)

**Table:**

N1—C1	1.1443 (16)	C4—H4	0.9500
C1—C2	1.4427 (16)	C5—C6	1.3893 (17)
C2—C3	1.3963 (16)	C5—H5	0.9500
C2—C7	1.4135 (16)	C6—C7	1.3957 (16)
C3—C4	1.3800 (17)	C6—H6	0.9500
C3—H3	0.9500	C7—C7^i^	1.488 (2)
C4—C5	1.3839 (18)		
			
N1—C1—C2	176.92 (12)	C4—C5—C6	120.68 (11)
C3—C2—C7	121.34 (11)	C4—C5—H5	119.7
C3—C2—C1	116.62 (10)	C6—C5—H5	119.7
C7—C2—C1	122.02 (10)	C5—C6—C7	121.38 (11)
C4—C3—C2	120.04 (11)	C5—C6—H6	119.3
C4—C3—H3	120.0	C7—C6—H6	119.3
C2—C3—H3	120.0	C6—C7—C2	116.99 (11)
C3—C4—C5	119.57 (11)	C6—C7—C7^i^	120.90 (12)
C3—C4—H4	120.2	C2—C7—C7^i^	122.09 (12)
C5—C4—H4	120.2		
			
C7—C2—C3—C4	−0.18 (18)	C5—C6—C7—C7^i^	−179.21 (9)
C1—C2—C3—C4	−178.65 (11)	C3—C2—C7—C6	0.51 (17)
C2—C3—C4—C5	−0.26 (19)	C1—C2—C7—C6	178.91 (11)
C3—C4—C5—C6	0.34 (19)	C3—C2—C7—C7^i^	179.27 (9)
C4—C5—C6—C7	0.02 (19)	C1—C2—C7—C7^i^	−2.34 (15)
C5—C6—C7—C2	−0.44 (17)		

Symmetry code: (i) −*x*+1, *y*, −*z*+1/2.

## References

[bb1] Brandenburg, K. (2010). *DIAMOND*. Crystal Impact GbR, Bonn, Germany.

[bb2] Bruker (2009). *APEX2*, *SAINT* and *SADABS*. Bruker AXS Inc., Madison, Wisconsin, USA.

[bb3] Furukawa, H., Kim, J., Ockwig, N. W., O’Keeffe, M. & Yaghi, O. M. (2008). *J. Am. Chem. Soc.* **130**, 11650–11661. 10.1021/ja803783c 18693690

[bb4] Paek, K., Knobler, C. B., Maverick, E. F. & Cram, D. J. (1989). *J. Am. Chem. Soc.* **111**, 8662–8671.

[bb5] Sheldrick, G. M. (2008). *Acta Cryst.* A**64**, 112–122. 10.1107/S0108767307043930 18156677

[bb6] Shimizu, S., Nakano, S., Kojima, A. & Kobayashi, N. (2014). *Angew. Chem. Int. Ed.* **53**, 2408–2412. 10.1002/anie.201310028 24478165

[bb7] Shimizu, S., Zhu, H. & Kobayashi, N. (2011). *Chem. Commun.* **47**, 3072–3074. 10.1039/c0cc05276k 21301724

[bb8] Wu, L.-L., Yang, C.-H., Sun, I.-W., Chu, S.-Y., Kao, P.-C. & Huang, H.-H. (2007). *Organometallics*, **26**, 2017–2023.

