# Supplementary figures and images for: Crystal structure of [1,1′-biphen­yl]-2,2′-dicarbo­nitrile
Source: Acta Crystallogr E Crystallogr Commun. 2015 May 30;71(Pt 6):o430. doi: 10.1107/S2056989015009561 (PMC4459381; doi:10.1107/S2056989015009561)

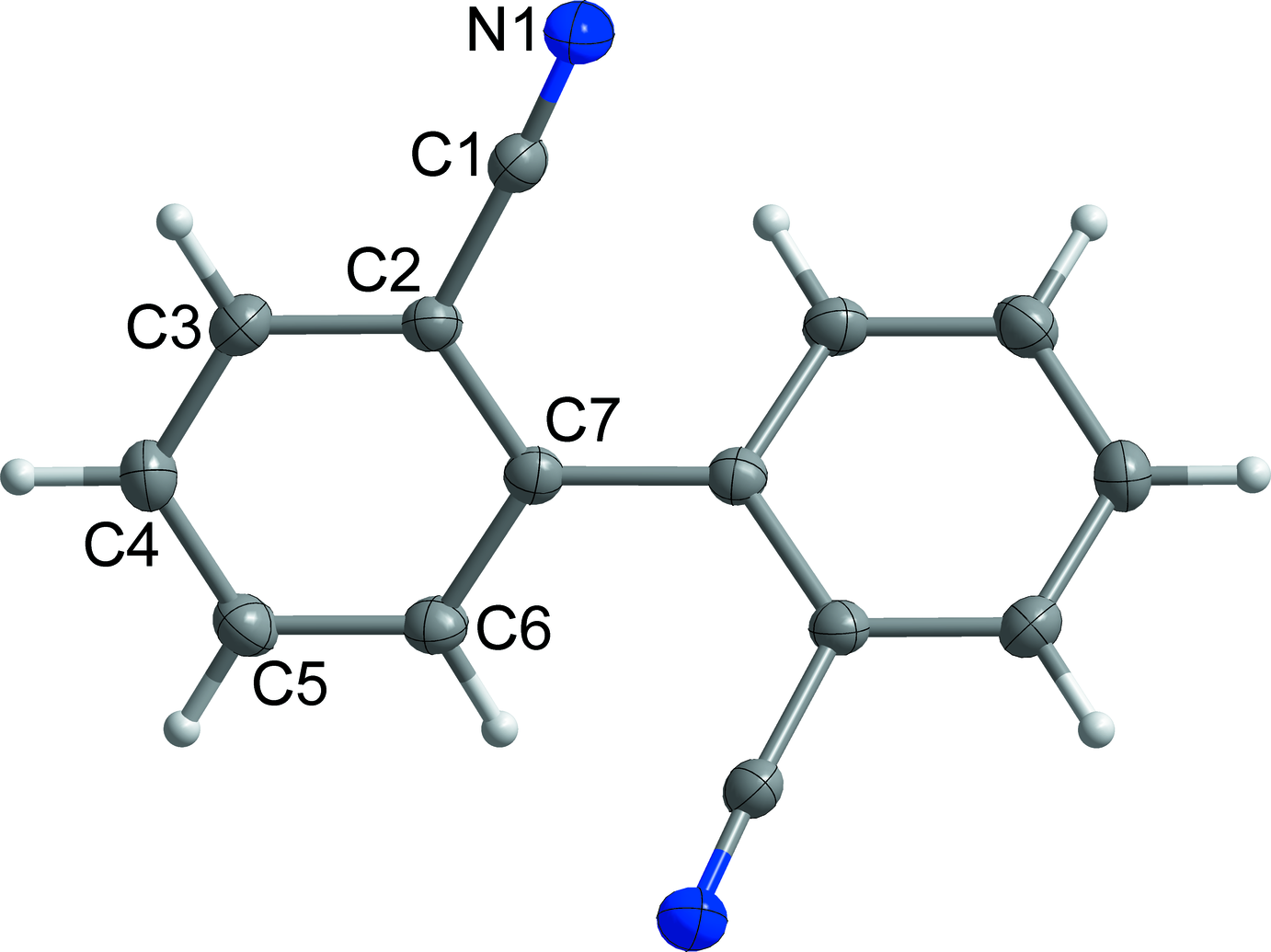

Supplement: Supplementary file 4 [file e-71-0o430-fig1.tif]

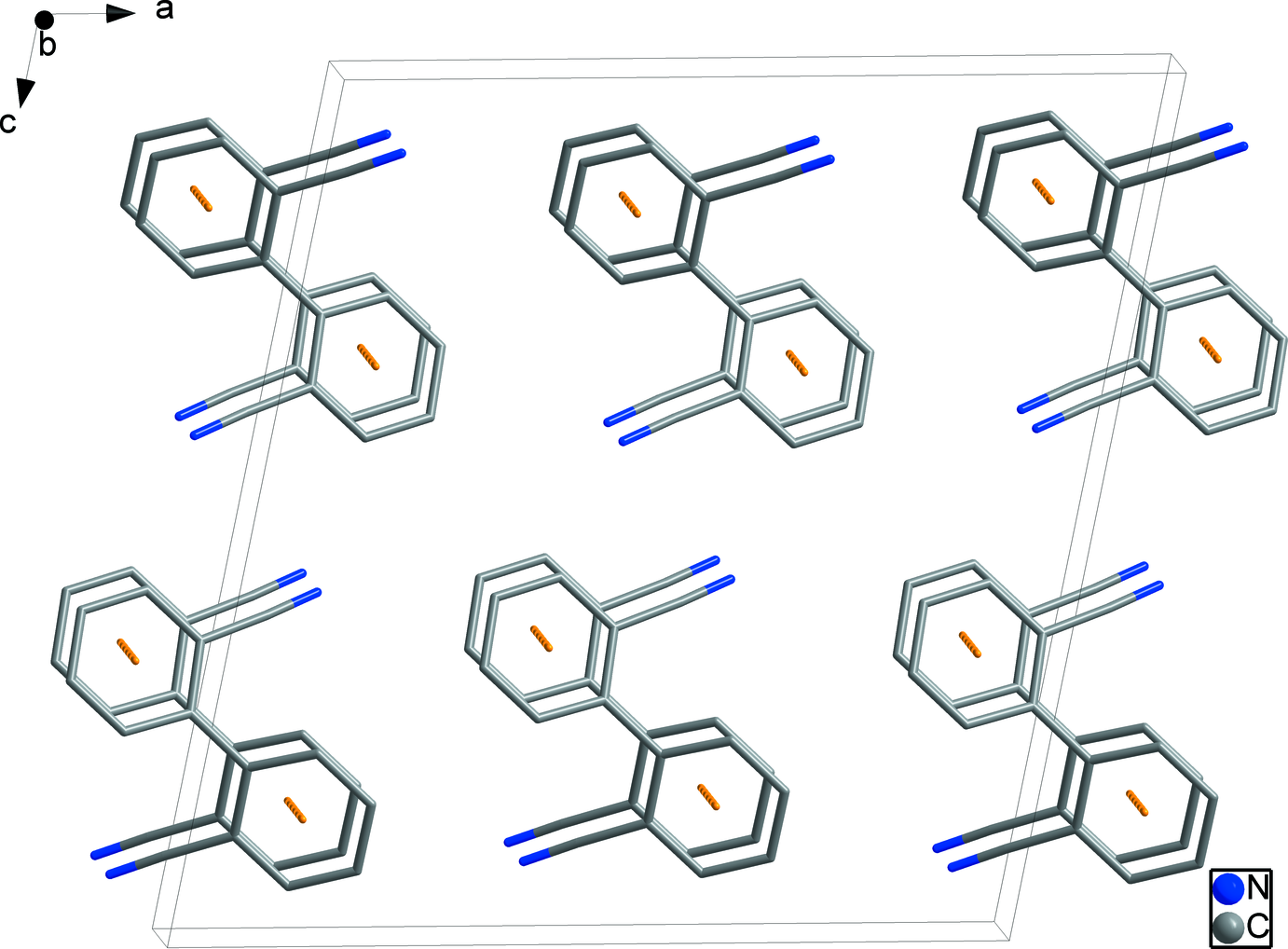

Supplement: Supplementary file 5 [file e-71-0o430-fig2.tif]
